# A Public HTLV-1 Molecular Epidemiology Database for Sequence Management and Data Mining

**DOI:** 10.1371/journal.pone.0042123

**Published:** 2012-09-10

**Authors:** Thessika Hialla Almeida Araujo, Leandro Inacio Souza-Brito, Pieter Libin, Koen Deforche, Dustin Edwards, Antonio Eduardo de Albuquerque-Junior, Anne-Mieke Vandamme, Bernardo Galvao-Castro, Luiz Carlos Junior Alcantara

**Affiliations:** 1 Gonçalo Moniz Research Center/Oswaldo Cruz Foundation, Salvador, Bahia, Brazil; 2 Bahia School of Medicine and Public Health/Bahia Foundation for Science Development, Salvador, Bahia, Brazil; 3 Rega Institute for Medical Research, Katholieke Universiteit Leuven, Leuven, Belgium; 4 Animal Models and Retroviral Vaccines Section, Center for Cancer Research, National Cancer Institute, National Institutes of Health, Bethesda, Maryland, United States of America; 5 MyBioData, Rotselaar, Belgium; 6 Centro de Malária e outras Doenças Tropicais, Instituto de Higiene e Medicina Tropical, Universidade Nova de Lisboa, Lisbon, Portugal; University of Oxford, United Kingdom

## Abstract

**Background:**

It is estimated that 15 to 20 million people are infected with the human T-cell lymphotropic virus type 1 (HTLV-1). At present, there are more than 2,000 unique HTLV-1 isolate sequences published. A central database to aggregate sequence information from a range of epidemiological aspects including HTLV-1 infections, pathogenesis, origins, and evolutionary dynamics would be useful to scientists and physicians worldwide. Described here, we have developed a database that collects and annotates sequence data and can be accessed through a user-friendly search interface. The HTLV-1 Molecular Epidemiology Database website is available at http://htlv1db.bahia.fiocruz.br/.

**Methodology/Principal Findings:**

All data was obtained from publications available at GenBank or through contact with the authors. The database was developed using Apache Webserver 2.1.6 and SGBD MySQL. The webpage interfaces were developed in HTML and sever-side scripting written in PHP. The HTLV-1 Molecular Epidemiology Database is hosted on the Gonçalo Moniz/FIOCRUZ Research Center server. There are currently 2,457 registered sequences with 2,024 (82.37%) of those sequences representing unique isolates. Of these sequences, 803 (39.67%) contain information about clinical status (TSP/HAM, 17.19%; ATL, 7.41%; asymptomatic, 12.89%; other diseases, 2.17%; and no information, 60.32%). Further, 7.26% of sequences contain information on patient gender while 5.23% of sequences provide the age of the patient.

**Conclusions/Significance:**

The HTLV-1 Molecular Epidemiology Database retrieves and stores annotated HTLV-1 proviral sequences from clinical, epidemiological, and geographical studies. The collected sequences and related information are now accessible on a publically available and user-friendly website. This open-access database will support clinical research and vaccine development related to viral genotype.

## Introduction

Human T-cell lymphotropic virus type 1 (HTLV-1) is the first described human retrovirus and was isolated from a patient with cutaneous T-cell lymphoma [1]. It is the causative agent of tropical spastic paraparesis/HTLV-1-associated myelopathy (TSP/HAM) [2], [3], adult T-cell leukemia/lymphoma (ATLL) [4], and other inflammatory diseases such as HTLV-1-associated infectious dermatitis [5], [6] and HTLV-1-associated uveitis (HAU) [7]. HTLV-1 is also associated with rheumatic diseases such as Sjögren's syndrome and rheumatoid arthritis [8], [9]. It is estimated that approximately 15 to 20 million people worldwide are infected with HTLV-1 [10]. Though epidemiological data show that HTLV-1 has a worldwide distribution [10], HTLV-1 infection is endemic in southwestern Japan [11], [12], sub-Saharan Africa [13], regions of the Caribbean [14], localized areas in Iran and Melanesia [15], and Brazil [16].

Since the discovery of HTLV-1 in 1980 [1], numerous studies of the virus have yielded sequence information from more than 2,000 unique isolates. Currently, no single resource is available to directly compare HTLV-1 sequence information with viral pathogenesis, transmission, gene polymorphisms, epidemiology, genotype-phenotype relationships, geographic distribution, and viral evolution. To further our understanding of the virus, we have developed a bioinformatic approach to catalogue, organize, and structure these annotated sequences into a comprehensible format. Bioinformatic systems use object-oriented or object-relational database models to store biological data and notes. These databases are designed to update, query, and retrieve information stored in the system [17]. By constructing a database to connect HTLV-1 genetic sequences and epidemiological data and organizing them into a relational database with a publically available and user-friendly web interface, we have created a novel resource for use in clinical research and vaccine development.

## Methods

### Collection and storage of data for the HTLV-1 Molecular Epidemiology Database

The HTLV-1 sequences and notes were taken directly from GenBank and submitted to the BLAST algorithm (Basic Local Alignment Search Tool) [18] in order to compare the information obtained from nucleotide sequences with those contained in the library or from the tool base itself. Data mining from collected sequences was performed to ensure information integrity, determine consistent patterns and systematic relationship between variables, and to remove unrelated sequences. Notes missing from GenBank were collected from their respective primary publications or from direct communication with the corresponding authors. Data were normalized and stored using Apache Webserver 2.1.6 and SGBD MySQL version 5.5. To facilitate the addition and manipulation of information, the IDE (Integrated Development Environment) MySQL-Front version 5.1 trial (http://www.mysqlfront.de/) was used. The database was initially modeled using MySQL and information was organized into a table containing the following fields: ID number, genomic region, status (complete or partial sequences), isolated, gender, age, ethnicity, geographic region, continent, clinical profile, proviral load, date of collection, CD4+, CD8+, sequence length, sequence, subtype, and subgroup.

### Structure of the HTLV-1 Molecular Epidemiology Database website

The website interfaces were developed in HTML and sever-side scripting written in PHP. The website interface contains specific search fields to allow various data combinations. User queries create a form (form tag) containing the values (variables) selected. This form generates a script that retrieves the data stored in the MySQL database. A second script organizes the data for display on the website, allowing for visualization of the information with the option to download the organized data. The developed database provides information regarding the indexed sequences in GenBank. The user is able to choose search criteria and perform a query to generate an output of relevant sequences and information. The sequence output may be downloaded in FASTA format and the information table in Microsoft Excel spreadsheets .xls format. The HTLV-1 Molecular Epidemiology database is hosted on the Gonçalo Moniz Research Center/Oswaldo Cruz Foundation Research Center server with access at http://htlv1db.bahia.fiocruz.br/.

## Results

Of the 2,457 sequences stored in the HTLV-1 Molecular Epidemiology Database, 1,933 (78.67%) sequences represent unique isolates. In addition, 91 (3.7%) sequences were added that did not contain isolate information in the GenBank notes. Sequences with unknown isolate information were analyzed by genomic region description and by country of origin. Sequences were identified as individual sequences by BLAST (identity <90%) and phylogenetic analysis (support <70%). Further, we reviewed sequences collected from GenBank and selected only those sequences that were relevant to the proposed database. We excluded clones and sequences from patients who had in the description the term “HTLV-1 or HTLV-I”, but were the result of another virus, such as HIV.

In total, 2,024 sequences were stored and made publically-accessible from the database. Of these sequences, 803 (39.67%) contain information about clinical status (TSP/HAM, 17.19%; ATL, 7.41%; asymptomatic, 12.89%; other diseases, 2.17%; and no information, 60.32%). In addition, 1,869 (92.8%) of sequences contain information about their geographic region of origin (Table 1). To determine the geographic origin of sequences, data was derived from either Genbank (73.5% of sequences) or from original publications and author correspondence (26.5% of sequences). Of the total sequences analyzed, 1,049 contained subtype information. This data was collected from Genbank (15.1% of sequences), articles (69% of sequences), and from LASP HTLV-1 subtyping Automated Tool (15.9% of sequences). In addition, 7.26% of sequences contain information on patient gender while 5.23% of sequences provide the age of the patient.

**Table 1:** Geographical distribution of collected HTLV-1 sequences by clinical profile.

		Clinical profile				
Geographic region	n (%)	TSP/HAM	ATL	Asymptomatic	Other Diseases	No information
Africa	311 (15.3)	20	12	63	7	209
Asia	612 (30.2)	219	83	108	18	184
Central America	53 (2.6)	10	3	9	12	19
South America	755 (37.3)	62	12	75	6	600
North America	30 (1.5)	4	10	0	0	16
Europe	59 (2.9)	8	7	2	0	42
Oceania	49 (2.4)	0	1	2	1	45
No information	155 (7.8)	25	22	2	0	106
**Total**	2,024	348 (17.19%)	150 (7.41%)	261 (12.89%)	44 (2.17%)	1221 (60.2%)

**Figure 1:**
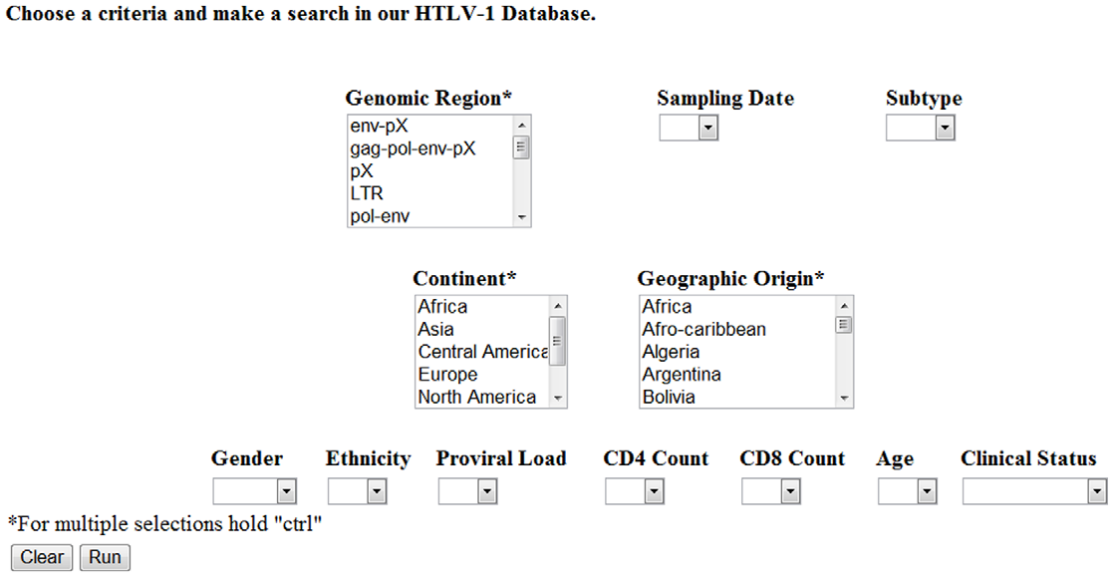
The HTLV-1 Molecular Epidemiology Database website interface. Search variables include genomic region, subtype, subgroup, sampling date, geographic origin, continent, age, gender, ethnicity, proviral load, CD4+ and CD8+ cell counts, and clinical status.

**Figure 2:**
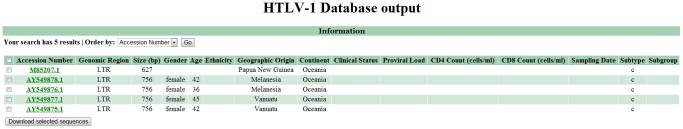
The HTLV-1 Molecular Epidemiology Database query results table. Retrieved information is posted as a table and the results may then be downloaded.

**Figure 3:**
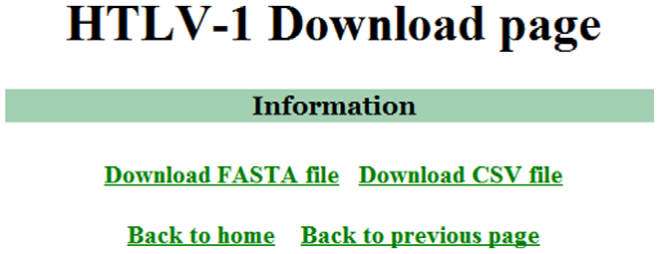
The HTLV-1 Molecular Epidemiology Database download page. Sequence results may be downloaded in FASTA or CSV file format.

The database is capable of searching stored HTLV-1 sequences by one or more specific criteria. The initial website interface contains variable fields to allow the user to refine the query output (Fig. 1). Current variables include genomic region, subtype, subgroup, geographic region of origin, and continent of origin. Additional variables include patient information such as age, gender, ethnicity, clinical status, proviral load, and CD4^+^ and CD8^+^ cell counts. Once the search variables are determined, the algorithm will retrieve the information and post the results as a table (Fig. 2). The results may then be downloaded in FASTA or CSV file format (Fig. 3). Currently, the HTLV-1 Molecular Epidemiology Database contains a Tutorial section and a map of the HTLV-1 open reading frames (ORFs) from the ATK-1 sequence [19]. These tools were designed to aid new users in developing advanced queries and interpreting returned results.

## Discussion

An important observation in the development of the database was the lack of complete information on the sequences in Genbank. There are variables in the HTLV-1 Molecular Epidemiology Database that are less complete than others, such as age, gender, and ethnicity. This is because the information is deficient in the publications and the authors have not made this information available. To fill the information gaps, we contacted the original authors and requested the missing data. Because of the difficulty in acquiring missing data, we encourage authors to provide more comprehensive information when submitting annotated sequences for publication in databases. These data are effective in making public health policies and for planning programs to combat the spread of infection.

Biological databases are essential tools to assist researchers in understanding, assessing, and comparing data generated from their research. Due to the large amount of knowledge created and the distribution of this information across different databases, it is difficult to ensure consistency and reliability of this data. The database described here proposes to aggregate information related to HTLV-1 sequences, thereby reducing redundancy and preserving data consistency, either from GenBank or related publications. We have developed a database of annotated HTLV-1 proviral sequences from clinical, epidemiological, and geographical studies that is accessible on a publically available and user-friendly website. This open-access database will support clinical research and vaccine development related to viral genotype. At present, the database contains more than 1,900 annotated sequences. We will provide regular updates to the database as new data becomes available. In the future we plan to incorporate new analysis tools into the website.
